# Poly (rC) binding protein 2 interacts with VP0 and increases the replication of the foot-and-mouth disease virus

**DOI:** 10.1038/s41419-019-1751-6

**Published:** 2019-07-04

**Authors:** Dan Li, Jing Zhang, Wenping Yang, Yanchun He, Yi Ru, Shaozu Fu, Lulu Li, Xiangtao Liu, Haixue Zheng

**Affiliations:** State Key Laboratory of Veterinary Etiological Biology and OIE/National Foot and Mouth Disease Reference Laboratory, Lanzhou Veterinary Research Institute, Chinese Academy of Agricultural Sciences, Lanzhou, Gansu China

**Keywords:** Immunology, Cell biology

## Abstract

Foot-and-mouth disease virus (FMDV) causes a highly contagious and debilitating disease in cloven-hoofed animals, which leads to devastating economic consequences. Previous studies have reported that some FMDV proteins can interact with host proteins to affect FMDV replication. However, the influence of the interactions between FMDV VP0 protein and its partners on FMDV replication remains unknown. In this study, we found that the overexpression of poly (rC) binding protein 2 (PCBP2) promoted FMDV replication, whereas the knockdown of PCBP2 suppressed FMDV replication. Furthermore, PCBP2 can interact with FMDV VP0 protein to promote the degradation of VISA via the apoptotic pathway. Further studies demonstrated that FMDV VP0 protein enhanced the formation of the PCBP2-VISA complex. Ultimately, we found that the degradation of VISA was weaker in PCBP2-knockdown and FMDV VP0-overexpressing cells, or FMDV VP0-knockdown cells than in the control cells. Summarily, our data revealed that the interaction between PCBP2 and VP0 could promote FMDV replication via the apoptotic pathway.

## Introduction

Foot-and-mouth disease virus (FMDV), a member of the *Aphthovirus* genus of the Picornaviridae family, is a pathogenic non-enveloped virus infecting cloven-hoofed animals^1,2^. FMDV, a positive-polarity and single-stranded RNA virus, encodes a single polyprotein processed into polypeptide products P1 (VP1–VP4), P2 (2A, 2B, and 2C), and P3 (3A, 3B, 3C, and 3D) by the three viral proteases L, 2A, and 3C^3^. It is widely accepted that the VP0 protein of enteroviruses is a cleavage precursor of VP2 and VP4^4^; however, the function of VP0 in FMDV replication remains unclear. FMDV exists as seven serotypes, and one serotype does not provide immunity against the others. This has contributed to the difficulty in the laboratory diagnosis and the control of foot-and-mouth disease^5^. Following the acute phase of FMDV infection in ruminants, some animals may experience prolonged asymptomatic persistent infection that can lead to genetic variation in the field and possibly results in the generation of new viral variants^4^. FMDV proteins could efficiently suppress cellular and organismal defenses, which are pivotal in establishing immune evasion^6–8^.

Viruses can be recognized by the host through pattern recognition receptors (PRRs), including Toll-like receptors (TLRs), RIG-I-like receptors (RLRs), Nod-like receptors (NLRs), and nucleic acid sensors^9,10^. Among the PRRs, RIG-I and MDA5 play key roles in sensing RNA virus invasion^11,12^. The C-terminal RNA helicase domains of RIG-I and MDA5 recognize viral RNAs, which induce an ATP-dependent conformational change that enables dimer or oligomer formation and exposes the caspase activation and recruitment domains (CARDs)^13–15^. The CARDs of RIG-I and MDA5 transmit signals to the downstream CARD-containing adaptor VISA (also known as MAVS, IPS-1, or Cardif)^9,16^. Previous studies have shown that poly (rC) binding protein 2 (PCBP2) belonging to a class of proteins that bind to poly (C) stretches of both RNA and DNA, recruits HECT-domain–containing E3 ligase AIP4 to polyubiquitinate, and degrades MAVS^17^. However, it is unclear whether PCBP2 regulates the replication of FMDV through VISA protein.

In this study, we found that PCBP2 interacts with FMDV VP0 protein. Overexpression of FMDV VP0 protein can enhance PCBP2-mediated degradation of VISA. Knockout of VISA increases the replication of FMDV. Our findings suggest that PCBP2 interacts with FMDV VP0 protein, which can increase PCBP2-mediated degradation of VISA and subsequently increase the FMDV replication.

## Materials and methods

### Cell lines, viruses, and antibodies

Human embryonic kidney (HEK293T) cells and the porcine kidney cell line (PK-15) (ATCC) were cultivated in Dulbecco’s modified eagle’s medium supplemented with 10% fetal bovine serum, 100 U penicillin/ml, and 100 μg streptomycin/ml in a humidified incubator with 5% CO_2_ at 37 °C. *Visa*^*–/*^^–^ and wild-type mouse embryonic fibroblasts (MEFs) were kindly provided by Dr. Hong-bing Shu (Wuhan University). The antibodies used in this study were as follows: rabbit ployclonal antibodies against PCBP2 (Abcam), IgG (Ig) (Sigma), and VISA (CST); mouse monoclonal antibodies against flag, Myc, IgG (Ig), β-actin, GST (Sigma), HA (OriGene), and GFP (Thermo Fisher Scientific). Mouse anti-VP3 sera were prepared in our laboratory using a recombinant FMDV VP3 protein. Sendai virus (SeV) inducing the activation of interferon was previously described^18^. The type O FMDV was propagated in PK-15 cells, and the supernatants of the infected cells were clarified and stored at –80 °C.

### Plasmid construction

Mammalian expression plasmids for Flag-, Myc-, HA-, GFP-, and GST-tagged porcine PCBP2 and its truncated mutants were constructed via standard molecular biology techniques. The PCBP2 (SP-AA) mutant was constructed by site-directed mutagenesis. FMDV expression plasmids for Flag-tagged 2B, 2C, 3A, 3C, 3D, VP0, VP2, and L were constructed as previously described^6^. In addition, the ISRE and IFN-β promoter luciferase reporter plasmids, pRL-TK plasmid and mammalian expression plasmids for HA-tagged VISA and CrmA were constructed as previously described^16,19,20^.

### Transfection and reporter gene assays

HEK293T cells (1 × 10^5^) were seeded on 48-well plates and transfected the following day via standard calcium phosphate precipitation. Meanwhile, an empty control plasmid was added to ensure that each transfection receives the same amount of total DNA. Here, 0.01 μg of pRL-TK Renilla luciferase reporter plasmid was added to each transfection to normalize the transfection efficiency. Luciferase assays were performed using a dual-specific luciferase assay kit (Promega), and the firefly luciferase activities were normalized on the based on *Renilla* luciferase activities.

### RNAi

Double-strand oligonucleotides corresponding to the target sequences were cloned into the pSuper. Retro RNAi plasmid (Oligoengine Inc.). The following sequences were targeted for porcine PCBP2 cDNA: PCBP2-RNAi #1, atcggttaagaagatgcgag; #2, gcacgtatcaacatctcaga; and #3, acagatctgcgtggtcatgt. The following sequences were targeted for FMDV VP0 cDNA: VP0-RNAi, ccaaacacctctggtcttga. The following sequences were targeted for GFP cDNA that were used as the control siRNA targeted sequences in the text: control-RNAi (coni), ggtgaaggtgatgctactta.

### Coimmunoprecipitation and immunoblotting analyses

These experiments were performed as previously described^16,19,21–24^. For transient transfection coimmunoprecipitation experiments, HEK293T cells were transfected with the appropriate plasmid. Twenty-four hours later, the cells were harvested and lysed in 1 ml of lysis buffer (20 mM Tris, pH 7.5, 150 mM NaCl, 1% Triton, 1 mM EDTA, 10 μg/ml aprotinin, 10 μg/ml leupeptin, and 1 mM phenylmethylsulfonyl fluoride). For each sample, 0.4 ml of cell lysate was incubated with 0.5 μg of the indicated antibody or control IgG and 40 μl of protein G agarose beads (Santa Cruz Biotechnology, Inc.) at 4 °C. After 4 h incubation, the beads were washed three times with 1 ml of lysis buffer containing 0.5 M NaCl. Afterward, the precipitates were analyzed by immunoblotting.

For endogenous coimmunoprecipitation experiments, PK-15 cells (5 × 10^7^) were infected with FMDV for the indicated time. The coimmunoprecipitation and immunoblotting experiments were performed as aforementioned.

### RNAi or overexpression-transduced stable PK-15 Cells

HEK293T cells were transfected with two packaging plasmids (pGAG-Pol and pVSV-G) combine with a control or PCBP2-RNAi, FMDV VP0-RNAi, and PCBP2-overexpression retroviral plasmid. Twenty-four hours later, the cells were incubated with fresh medium without antibiotics for another 24 h. The recombinant virus-containing medium was filtered and added to PK-15 cells in the presence of polybrene (8 μg/ml). The infected cells were selected with puromycin (0.5 μg/ml) for 7 days before additional experiments.

### Real-time PCR analysis

PK-15 cells were infected with FMDV at 0.1 of MOI. Subsequently, total RNA was isolated from cells utilizing the TRIzol reagent (TaKaRa) and subjected to real-time PCR analysis to evaluate the viral genome copies of FMDV using iQ SYBR Green Supermix and C1000 Thermal Cycler (Bio-Rad). Gene-specific primer sequences were as follows: *VP0* sense: 5′-ggcaacaccgggagtatcat-3′, *VP0* anti-sense: 5′-tgactatggtccaggcacag-3′; *PCBP2* sense: 5′-gcgcctgcagtttttggc-3′, *PCBP2* anti-sense: 5′-gtagccggatggtgagagtg-3′; and *GAPDH* sense: 5′-actcactcttctacctttgatgct-3′, *GAPDH* anti-sense: 5′-tgttgctgtagccaaattca-3′.

The quantification of genome copies of FMDV was performed as previously described^25^.

### Apoptosis assay by flow cytometry

Apoptosis assay was carried out to identify and quantify cells apoptosis utilizing Annexin V-FITC/PI apoptosis detection kit (Beyotime Institute of Biotechnology). HEK293T cells were plated in a six-well plate for 12 h, and then transfected with the indicated expression plasmids (1 μg) for 24 h. Cells were collected by trypsinization, washed twice with PBS and centrifuged at 500×*g*, room temperature for 5 min. Then the cells were suspended in 500 μl PBS and incubated with 5 μl Annexin V‑FITC (Annexin V‑FITC Apoptosis Detection Kit; Beyotime Institute of Biotechnology, Haimen, China), and 10 μl (20 μg/ml) propidium iodide (PI) solution (Beyotime Institute of Biotechnology) at room temperature for 20 min in the dark. Further, the samples were measured using a flow cytometer.

### Statistical analysis

The significance of differences between samples was assessed using an unpaired two-tailed Student *t* test. The variance was estimated by calculating the standard deviation (SD) and represented by error bars. All experiments were performed independently at least three times, with a representative experiment being shown. *p < 0.05, **p < 0.01. Densitometry quantification was analyzed with ImageJ Software.

## Results

### PCBP2 potentiates FMDV replication

**Fig. 1:**
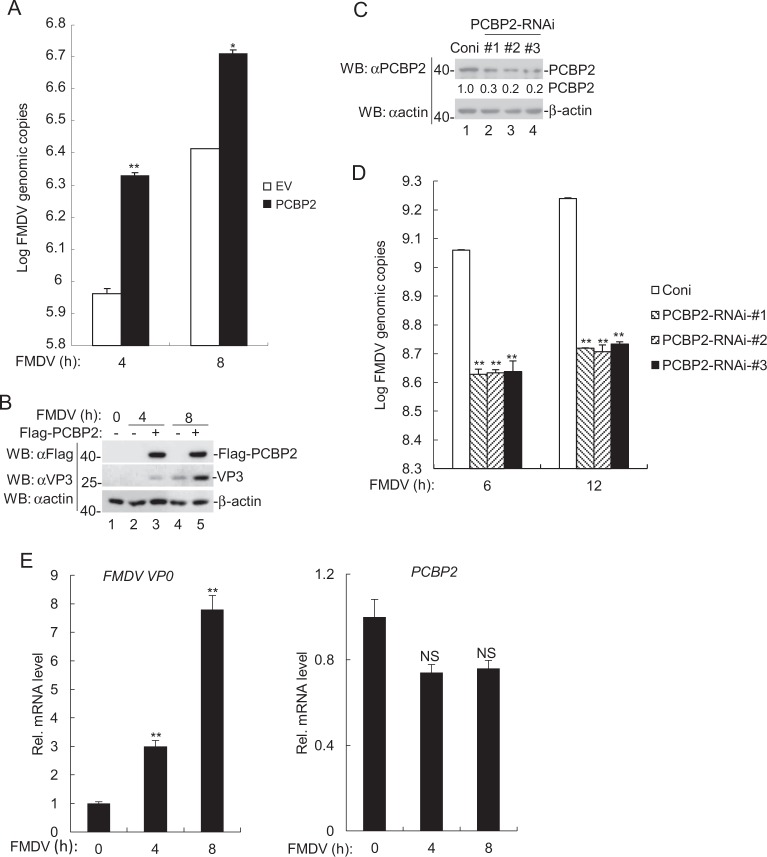
PCBP2 potentiates FMDV replication. **a** PCBP2 increased FMDV replication in PCBP2-overexpressing PK-15 cells. PCBP2-overexpressing PK-15 cells were infected with FMDV (MOI = 0.1) for 4 and 8 h, and the copy number of FMDV RNA was evaluated by RT-PCR. **b** Western immunoblotting analysis of FMDV VP3 protein levels in PCBP2-overexpressing PK-15 cells. The experiments were similar to those described in **a**. Western immunoblotting analysis with the antibodies against flag, VP3, and β-actin proteins. **c** Western immunoblotting analysis of PCBP2 protein levels in PCBP2-knockdown PK-15 cells. Western immunoblotting analysis with the antibodies against PCBP2 and β-actin proteins. Degree of PCBP2-knockdown was calculated by Image J software. **d** PCBP2 reduced FMDV replication in PCBP2-knockdown PK-15 cells. The experiments were similar to those described in **a**. **e** Effects of FMDV on the transcription of FMDV VP0 and PCBP2 genes. PK-15 cells were infected with FMDV (MOI = 0.1) for 0, 4, and 8 h, and the mRNA levels of VP0 and PCBP2 were analyzed by RT–PCR. The data represent the mean ± SD of the three independent experiments (*p < 0.05; **p < 0.01; NS, not significant, *p*> 0.05). EV empty vector, Coni control-RNAi

To evaluate the role of PCBP2 in FMDV replication, the viral yield was examined in PCBP2-overexpressing PK-15 cells. The RT-PCR results showed that the copy number of FMDV RNA was significantly upregulated in PCBP2-overexpressing PK-15 cells, compared to the mock cells transfected with an empty vector (Fig. 1a). Moreover, the expression of FMDV VP3 protein increased in PCBP2-overexpressing PK-15 cells (Fig. 1b). Next, we investigated the function of endogenous PCBP2 in FMDV replication. The immunoblotting results presented that the expression of endogenous PCBP2 decreased in PK-15 cells transfected with three PCBP2-RNAi plasmids (Fig. 1c), and PCBP2 knockdown dramatically reduced the copy number of FMDV RNA (Fig. 1d).

To explore whether FMDV infection affects the synthesis of PCBP2 RNA, PK-15 cells were infected with FMDV followed by real-time RT-PCR experiment. We observed that the transcription of FMDV *VP0* genes markedly increased, whereas the transcription of *PCBP2* genes showed no differences between FMDV-infected PK15 cells and control cells (Fig. 1e). Collectively, these results suggest that PCBP2 overexpression is able to increase the replication of FMDV.

### PCBP2 interacts with FMDV proteins

**Fig. 2:**
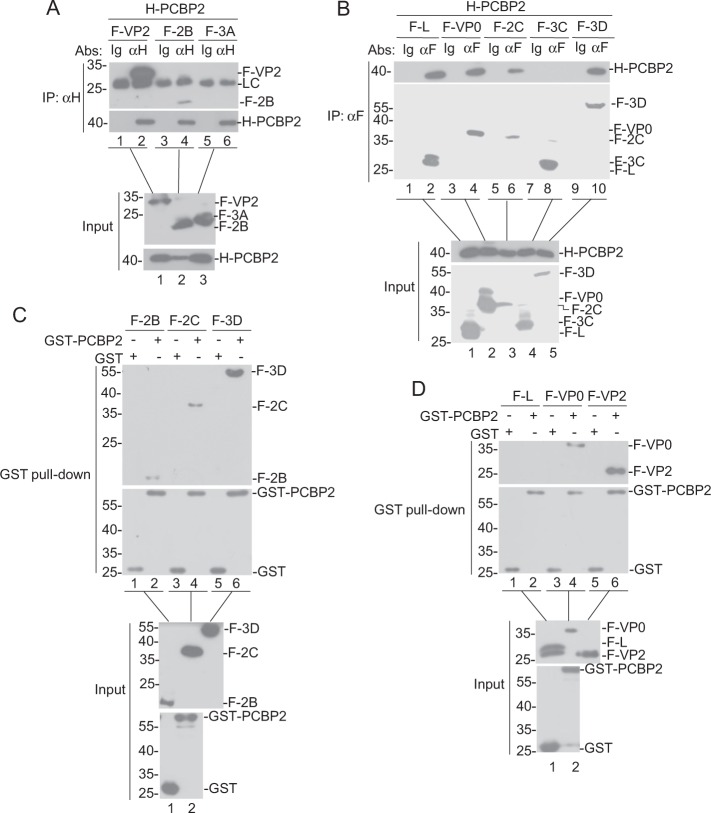
PCBP2 interacts with FMDV proteins. **a**, **b** The interaction between PCBP2 and FMDV proteins in mammalian overexpression systems was identified by coimmunoprecipitation experiment. The HEK293T cells (2 × 10^6^) were transfected with HA-PCBP2 plasmid or expressing Flag-tagged VP2, 2B, 3A, L, VP0, 2C, 3C, and 3D proteins (5 μg each) for 24 h. Afterward, PCBP2 and FMDV proteins were analyzed by coimmunoprecipitation and immunoblotting analyses using the antibodies against flag and HA. **c**, **d** GST pull-down assay. Glutathione beads conjugated with GST or with GST-PCBP2 fusion protein were incubated with recombinant FMDV proteins. After washing three times, the proteins were eluted from the beads, and SDS-PAGE was performed. The presence of FMDV proteins was detected via immunoblotting utilizing the anti-flag monoclonal antibody. GST and GST-PCBP2 protein expression was confirmed via immunoblotting using the rabbit anti-GST polyclonal antibody. F flag tag, H HA tag, LC light chain

To explore the mechanism of PCBP2 overexpression promoting FMDV replication, the transient transfection and coimmunoprecipitation assays were carried out. As shown in Fig. 2a, b, PCBP2 could interact with VP2, 2B, L, VP0, 2C, and 3D proteins of FMDV, but not with 3A and 3C. To determine whether PCBP2 directly binds to VP2, 2B, L, VP0, 2C, and 3D proteins of FMDV, we performed GST pull-down assays. The results showed that PCBP2 binds to VP2, 2B, VP0, 2C, and 3D proteins of FMDV, but not to L (Fig. 2c, d). These results indicate that PCBP2 can directly interact with the VP2, 2B, VP0, 2C, and 3D proteins of FMDV.

### Porcine PCBP2 inhibits SeV-triggered signaling

**Fig. 3:**
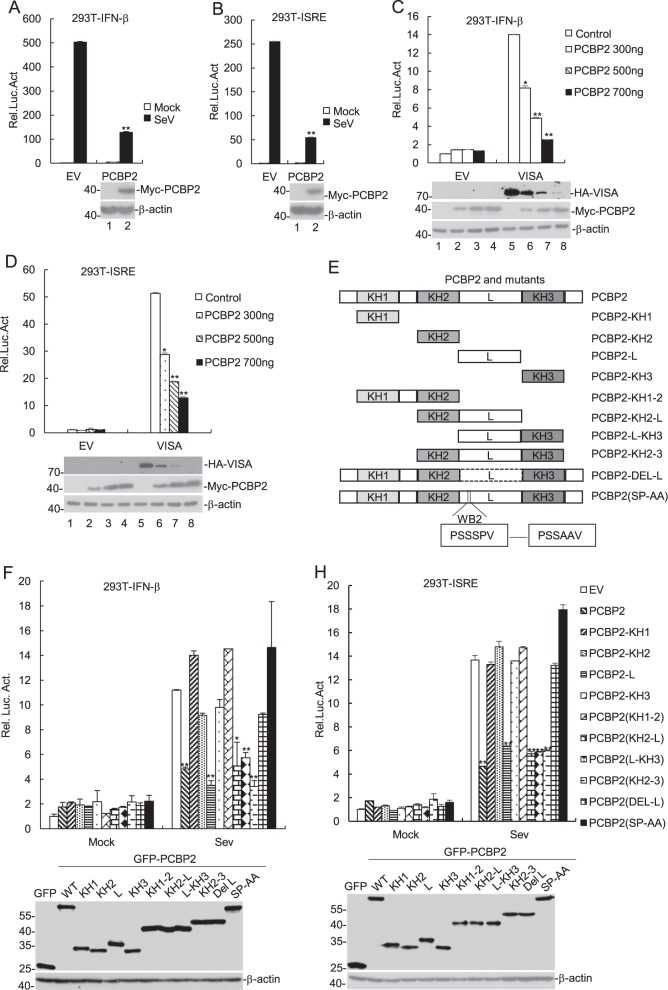
PCBP2 inhibits IFN-β signaling pathway. **a**, **b** The effects of PCBP2 overexpression on SeV-triggered IFN-β promoter and ISRE activation were evaluated. HEK293T cells (1 × 10^5^) were transfected with IFN-β reporter or ISRE (0.1 μg) and with PCBP2 expression (0.1 μg) plasmids for 24 h. The cells were then infected with SeV for 12 h or were uninfected (left) before luciferase assays were performed. EV empty vector. **c**, **d** Dose-dependent effects of PCBP2 on VISA-triggered activation of IFN-β promoter and ISRE were assessed. The experiments were performed similar to those described in **a**. EV empty vector. **e** Panel shows schematic representations of PCBP2 proteins. **f**, **h** The sites or domains of PCBP2 inhibit IFN-β signaling pathway. The experiments were performed similar to those described in **a**. EV, empty GFP vector. The data represent the mean ± SD of the three independent experiments (*p < 0.05; **p < 0.01). Luc luciferase

A previous study has demonstrated that human PCBP2 inhibits the production of interferons (IFNs)^17^. The human PCBP2 consists of 335 aa residues and shares 99% sequence identity with porcine PCBP2. Hence, we speculated that porcine PCBP2 is likely to affect interferon production. Here, we first investigated the function of porcine PCBP2 in SeV-triggered type I IFN production. The reporter assay results indicated that the overexpression of porcine PCBP2 inhibited SeV-triggered activation of the IFN-β promoter and ISRE (an IRF3-binding motif) (Fig. 3a, b). Further experiments showed that porcine PCBP2 overexpression also inhibited VISA-triggered activation of IFN-β promoter and ISRE in a dose-dependent manner (Fig. 3c, d). In addition, we found that porcine PCBP2 degraded VISA in a dose-dependent manner (Fig. 3c, d). Collectively, these data suggest that porcine PCBP2 inhibits the VISA-triggered signaling pathway.

Previously, it has been revealed that the linker region and WB2 SP residues of human PCBP2 were essential for the inhibition of innate antiviral immunity^17^. To compare the function of porcine PCBP2 with human PCBP2, we generated a series of different truncated porcine PCBP2 and a point substitution of the SP amino acid motif in the WB2 region (Fig. 3e). As shown in Fig. 3f–h, the WB2 SP motif and the linker region of porcine PCBP2 suppressed SeV-triggered activation of IFN-β promoter and ISRE. These data show that the WB2 SP motif and the linker region of porcine PCBP2 play crucial role in inhibiting SeV-triggered signaling pathway.

### The 2C, 2B, 3D, and VP0 proteins of FMDV synergize with porcine PCBP2 to inhibit VISA-triggered signaling

**Fig. 4:**
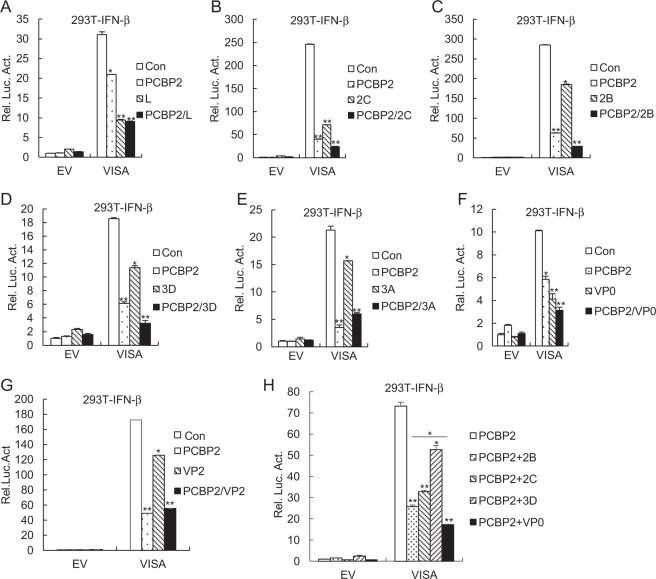
Effects of FMDV proteins synergized with PCBP2 on VISA-triggered signaling. **a**–**g** Effects of L **a**, 2C **b**, 2B **c**, 3D **d**, 3A **e**, VP0 **f**, and VP2 **g** proteins of FMDV synergized with PCBP2 on VISA-triggered activation of IFN-β promoter. HEK293T cells (1 × 10^5^) were transfected with IFN-β reporter (0.1 μg) and the indicated expression (0.1 μg) plasmids for 24 h before luciferase assays were performed. **h** Effects of 2B, 2C, 3D, and VP0 proteins of FMDV synergized with PCBP2 on VISA-triggered activation of IFN-β promoter. The experiments were performed similar to those described in **a**. The data represent the mean ± SD of the three independent experiments (*p < 0.05; **p < 0.01). EV empty vector, Luc luciferase, Con control

Recent studies have illustrated that 3A and VP3 proteins of FMDV are able to inhibit interferon signaling pathway^6–8^. To screen the FMDV proteins synergizing with PCBP2 to inhibit IFN-β signaling pathway, HEK293T cells were transfected with plasmids encoding FMDV proteins and PCBP2. We observed that 2C, 2B, 3D, and VP0 proteins of FMDV synergized with PCBP2 to suppress VISA-triggered activation of the IFN-β promoter (Fig. 4a–d), whereas L, 3A, and VP2 proteins of FMDV did not have the synergy (Fig. 4e–g). Further, our study revealed that the inhibitory effect of PCBP2 combined with VP0 protein on VISA-triggered activation of IFN-β promoter was superior to those of PCBP2 combined with individual 2B, 2C, and 3D proteins of FMDV (Fig. 4h). These results suggest that 2C, 2B, 3D, and VP0 proteins of FMDV synergize with PCBP2 to inhibit VISA-triggered signaling pathway.

### The VP0 protein of FMDV enhances the interaction between PCBP2 and VISA

**Fig. 5:**
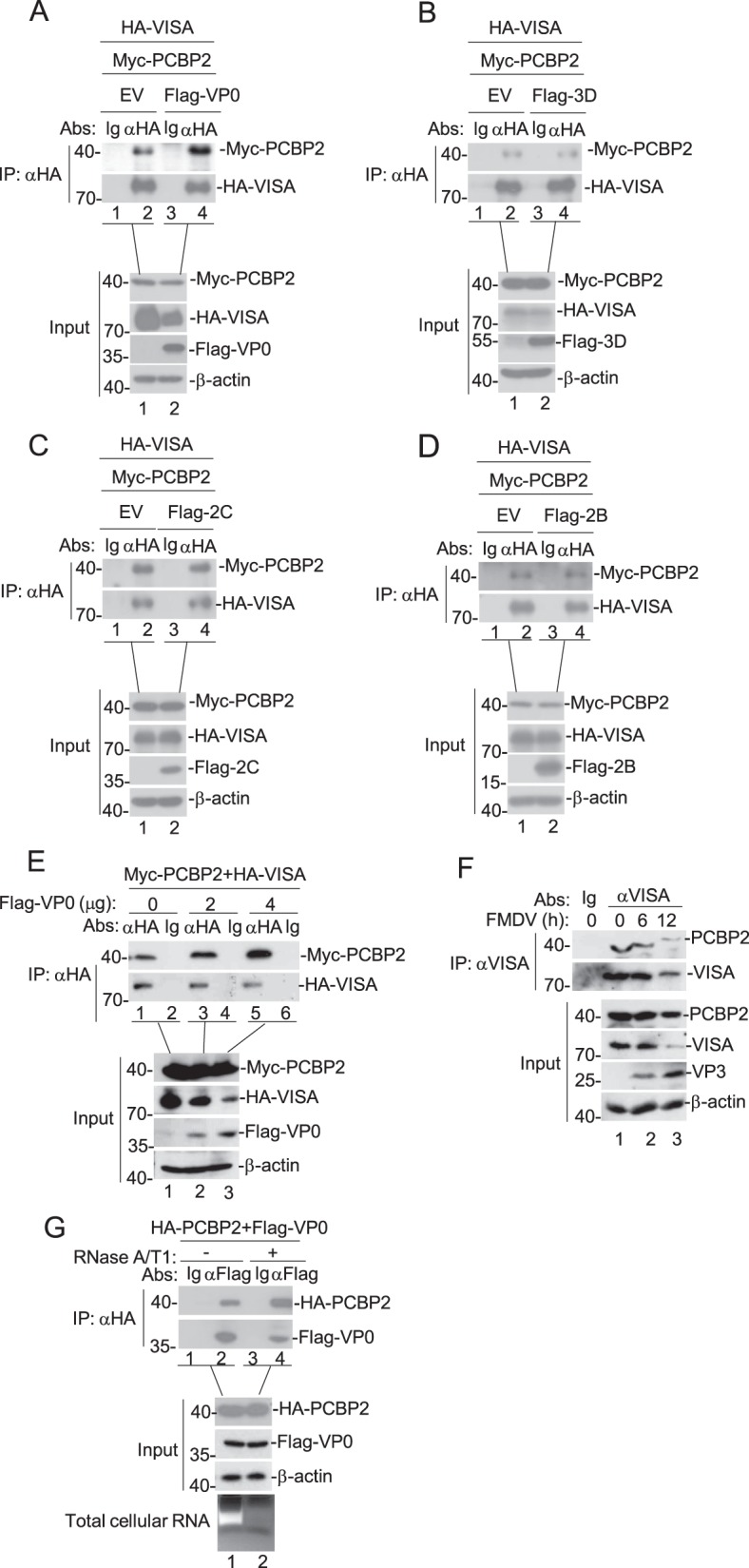
Effects of overexpression of FMDV proteins on the interaction between PCBP2 and VISA. **a**–**d** HEK293T cells (2 × 10^6^) were transfected with the indicated plasmids (5 μg each) for 24 h. Coimmunoprecipitation and immunoblotting analyses were performed with the indicated antibodies. **e** Dose-dependent effects of FMDV VP0 protein on the interaction between VISA and PCBP2. The experiments were performed similar to those described in **a**. **f** Effects of FMDV on the interaction between VISA and PCBP2. PK-15 cells (5 × 10^7^) were infected with FMDV (MOI = 0.1) for 0, 6, and 12 h. Coimmunoprecipitation experiments were performed with antibody against VISA, and the immunoprecipitates were analyzed by immunoblotting using the antibodies against PCBP2 and VISA protein. The lysates were analyzed by immunoblotting with the indicated antibodies. **g** Effects of RNase A/T1 treatment on PCBP2–VP0 interaction. Extractions of HEK293T cells overexpressing different proteins were treated or not treated with RNases A/T1 for 1 h before coimmunoprecipitation and immunoblotting with the indicated antibodies. Ethidium bromide-stained agarose gels showed the total amount of RNA present. EV empty vector

Considering that the association between PCBP2 and VISA could regulate RIG-I- like helicase signaling^17^, we further determined whether the expression of 2C, 2B, 3D, and VP0 proteins of FMDV had an effect on the interaction between PCBP2 and VISA. In transient transfection and coimmunoprecipitation experiments, we observed that the VP0 protein of FMDV promoted the interaction between PCBP2 and VISA (Fig. 5a), whereas 3D, 2C, and 2B proteins of FMDV did not exhibit this ability (Fig. 5b–d). In addition, we found that the VP0 protein of FMDV induced VISA degradation (Fig. 5a). Further experiments indicated that the VP0 protein of FMDV promoted the interaction between PCBP2 and VISA in a dose-dependent manner in HEK293T cells (Fig. 5e). To examine whether PCBP2 interacts with VISA in physiological conditions, endogenous coimmunoprecipitation experiments were conducted. As shown in Fig. 5f, PCBP2–MAVS interaction decreased after FMDV infection in PK-15 cells. The VP0–PCBP2 interaction might be mediated by the formation of nonspecific RNA bridge, as PCBP2 has a strong affinity for nucleic acids. However, RNase A/T1 treatment of cell extracts before coimmunoprecipitation did not reduce VP0–PCBP2 association, indicating that the interaction is not affected by the nonspecific RNA-mediated effects (Fig. 5g). These data indicate that the VP0 protein of FMDV enhances the interaction between PCBP2 and VISA.

### The VP0 protein of FMDV synergizes with PCBP2 to promote VISA degradation

**Fig. 6:**
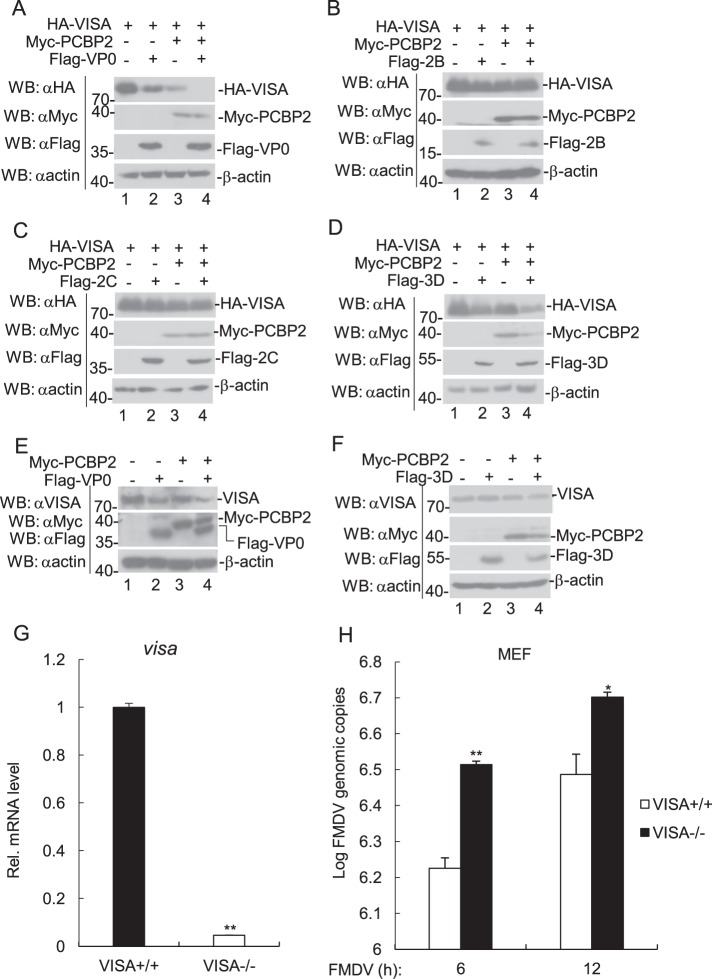
Effects of overexpression of PCBP2 and FMDV proteins on the expression level of VISA. **a**–**d** Effects of overexpression of PCBP2 and FMDV VP0, 2B, 2C, and 3D on the expression level of VISA. HEK293T cells (5 × 10^5^) were transfected with the indicated expression plasmids (2 μg each) for 24 h. Immunoblotting analyses were performed using the indicated antibodies. **e**–**f** Effects of overexpression of PCBP2 and FMDV VP0 and 3D on the expression level of endogenous VISA. The experiments were performed similar to those described in **a**. **g** RT-PCR was used to detect the transcription of VISA in VISA-deficient MEF cells. **h** Deficiency of VISA increases FMDV replication in MEF cells. VISA-deficient MEF cells were infected with FMDV (MOI = 0.1) for the indicated time. RT-PCR analysis was carried out to evaluate FMDV replication. The data represent the mean ± SD of the three independent experiments (*p < 0.05; **p < 0.01)

As shown in Fig. 5a, we found that the VP0 protein of FMDV induced VISA degradation. Next, we analyzed the effect of the VP0 protein of FMDV on PCBP2-mediated VISA degradation. The transient transfection results showed that the VP0 protein of FMDV promoted PCBP2 mediated VISA degradation (Fig. 6a), whereas 2B, 2C, and 3D proteins did not have this ability (Fig. 6b–d). In agreement with the results of exogenous coimmunoprecipitation, the VP0 protein of FMDV promoted PCBP2-mediated degradation of endogenous VISA (Fig. 6e); however, FMDV 3D did not significantly promoted the degradation of endogenous VISA mediated by PCBP2 (Fig. 6f). Therefore, the present study mainly focused on investigating the function of the cellular protein PCBP2 and FMDV VP0. These data collectively indicate that the VP0 protein of FMDV synergizes with PCBP2 to promote the degradation of VISA.

This suggests that VISA had an effect on FMDV replication. Here, VISA-deficient MEFs were used to further examine the roles of VISA in FMDV replication. The Q-PCR results presented that the mRNA level of VISA significantly decreased in VISA-deficient cells compared to the control cells (Fig. 6g), and the replication of FMDV increased in VISA deficient MEFs compared to the control cells (Fig. 6h). Altogether, these results showed that VISA plays a pivotal role in the regulation of FMDV replication.

### The VP0 protein of FMDV and PCBP2 induce VISA degradation via the apoptotic pathways

**Fig. 7:**
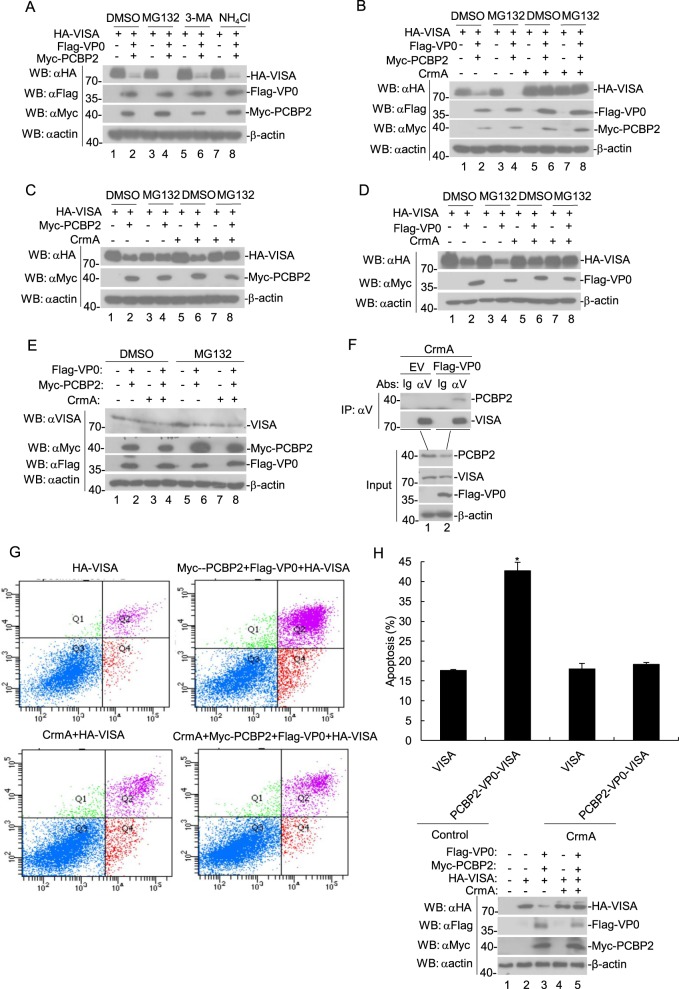
FMDV VP0 coexpresses with PCBP2-mediated degradation of VISA via the apoptotic pathway. **a**–**d** Coexpression of PCBP2 and FMDV VP0 degraded VISA via the apoptotic pathway. HEK293T cells (5 × 10^5^) were transfected with the indicated expression plasmids (2 μg each) for 18 h and then treated with dimethyl sulfoxide (DMSO), MG-132, 3-MA, or NH_4_Cl for 6 h. Immunoblotting analyses were performed using the indicated antibodies. **e** Coexpression of PCBP2 and FMDV VP0 degraded endogens VISA via the apoptotic pathway. The PK-15 cells (5 × 10^5^) were transfected with the indicated expression plasmids (4 μg each) for 18 h and then treated with dimethyl sulfoxide (DMSO), and MG-132 for 6 h. Immunoblotting analyses were performed using the indicated antibodies. **f** FMDV VP0 increases the interaction between endogenous PCBP2 and VISA. The PK-15 cells (2 × 10^6^) were transfected with the indicated plasmids (8 μg each) for 24 h. Coimmunoprecipitation and immunoblotting analyses were performed using the indicated antibodies. **g** Effects of VP0 and PCBP2 on the apoptosis of HEK293T cells. HEK293T cells were transfected with the indicated plasmids (1 μg) for 24 h. The cellular apoptosis rate was measured by flow cytometry. **h** Quantification of the results shown in G. Western blotting was used to analyze protein expression of VISA, VP0, and PCBP2. αV anti-VISA, EV empty vector

To investigate the mechanisms of VP0 and PCBP2 on the stability of VISA, transiently transfected HEK293T cells were treated with inhibitors targeting various protein degradation pathways. The proteasome inhibitor (MG132), the lysosome inhibitor ammonium chloride (NH_4_Cl), or the autophagosome inhibitor 3-methyladenine (3-MA) hardly inhibited the degradation of VISA in cells transfected with VP0, PCBP2, and VISA (Fig. 7a). This indicates that VP0 and PCBP2 induce the degradation of VISA through a different pathway. Afterward, we found that the apoptosis inhibitor CrmA markedly inhibited the degradation of VISA in cells transfected with VP0, PCBP2, and VISA (Fig. 7b), suggesting that VP0 and PCBP2 induces the degradation of VISA through the apoptotic pathway. In addition, MG132, but not CrmA, obviously inhibited the degradation of VISA in cells transfected with PCBP2 and VISA (Fig. 7c). Analogously, CrmA, but not MG132, markedly inhibited the degradation of VISA in cells transfected with VP0 and VISA (Fig. 7d). Furthermore, we observed that CrmA suppressed the degradation of endogenous VISA in cells transfected with VP0 and PCBP2 (Fig. 7e). Co-immunoprecipitation experiments showed that FMDV VP0 protein enhanced the interaction between endogenous VISA and PCBP2 (Fig. 7f). Subsequently, the flow cytometry analysis was performed to confirm the effects of VP0, PCBP2, and CrmA on the apoptosis of cells. The flow cytometry data presented that CrmA obviously suppressed the rate of apoptosis of cells transfected with VP0, PCBP2, and VISA, whereas they did not affect the rate of apoptosis of cells transfected with VISA individually (Fig. 7g, h). These results suggest that the VP0 protein of FMDV and PCBP2 induce VISA degradation via the apoptotic pathway.

### Endogenous PCBP2 and FMDV VP0 play a crucial role in the degradation of VISA

**Fig. 8:**
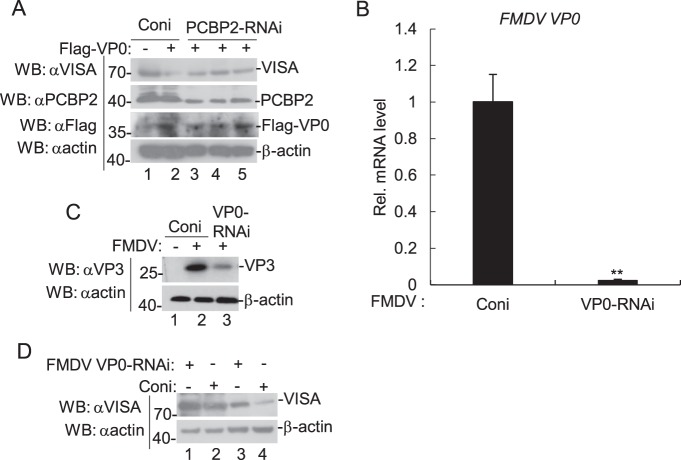
Effect of endogenous PCBP2 and FMDV VP0 on the expression of VISA. **a** Effects of overexpression of FMDV VP0 on the expression of VISA in PCBP2-knockdown PK-15 cells. The PCBP2-knockdown PK-15 cells (5 × 10^5^) were transfected with the indicated plasmids (2 μg each) for 24 h. Then, the immunoblotting analyses were performed utilizing the indicated antibodies. **b** Effects of VP0-RNAi plasmids on the transcription of FMDV VP0. The VP0-knockdown PK-15 cells were infected with FMDV (MOI = 0.1) for 12 h before real-time RT–PCR experiments were performed. **c** Effects of knockdown of FMDV VP0 on the expression of FMDV VP3 protein. The experiments were performed similar to those described in **b**. **c** Effects of knockdown of FMDV VP0 on the expression level of VISA. The VP0- knockdown PK-15 cells were infected with FMDV (MOI = 0.1) for 12 h or uninfected followed by immunoblotting analyses were performed with the indicated antibodies. The data represent the mean ± SD of the three independent experiments (**p < 0.01). Con control

To explore further the effect of endogenous PCBP2 on the degradation of VISA mediated by FMDV VP0 protein, we detected the expression of VISA in PCBP2-knockdown PK-15 cells. The immunoblotting results showed that the overexpression of FMDV VP0 weakened the degradation of VISA in PCBP2-knockdown PK-15 cells lines (Fig. 8a). To investigate the role of endogenous FMDV VP0 in the degradation of VISA, RNAi plasmids for FMDV VP0 were constructed. We found that RNAi plasmids could markedly reduce the transcription of endogenous FMDV VP0 in FMDV-infected PK-15 cells (Fig. 8b). In addition, we observed that the expression of FMDV VP3 protein reduced in VP0-knockdown FMDV-infected PK-15 cells compared to those in the control cells (Fig. 8c). Furthermore, the immunoblotting analysis results showed that the expression level of VISA increased in FMDV VP0-knockdown FMDV-infected PK-15 cells compared to those in the control (Fig. 8d). These findings demonstrate that PCBP2 cooperates with FMDV VP0 protein in the degradation of VISA.

## Discussion

It was previously reported that Picornaviruses are able to utilize the functions of PCBP2 proteins to promote viral translation and RNA replication, thereby contributing to the replication of viruses^26^. Considering that FMDV is a member of the Picornavirus family^27^, we speculated that PCBP2 is likely to play a vital role in FMDV replication. However, the molecular mechanism by which PCBP2 mediates FMDV replication remains unclear. A study has demonstrated that human PCBP2–VISA pathways are able to mediate the innate immune responses to viruses^17^. In this study, we hypothesized that FMDV proteins target the PCBP2–VISA axis to evade the innate immune responses. Here, we identified that the VP0 protein of FMDV acts as a negative regulator of PCBP2/VISA-mediated induction of downstream antiviral genes and innate antiviral response.

Several lines of evidence have suggested that porcine and human PCBP2 exhibit the same function in the inhibition of innate antiviral response. First, porcine PCBP2, consisting of 335 amino acids, is highly homologous with human PCBP2 (99%). Second, porcine PCBP2 also inhibited SeV-triggered activation of IFN-β promoter and ISRE. Third, the SP residues of the WB2 region and the linker region of porcine PCBP2 play an important role in inhibiting the IFN-β signaling pathway.

It is widely accepted that FMDV 3A and VP3 suppressed the IFN-β signaling pathway by reducing mRNA level of VISA^6,7^. Here, we found that VP0 inhibited IFN-β signaling pathway by enhancing PCBP2-mediated degradation of VISA. Moreover, FMDV protease L protein not only mediates the degradation of factor kappa B (NF-κB) subunit p65/RelA^28^, but also correlates with the degradation of IRF-3/7 (interferon regulatory factor 3/7), both of which play important roles in the production of IFN^29^. Consistently, it has been demonstrated that FMDV protease 3C protein can cleave nuclear transcription NF-κB essential modulator (NEMO), and thus impair the ability of NEMO to activate the downstream of IFN production as well as to participate in the MDA-5/RIG-I pathway^30^. FMDV VP0 and PCBP2 degrade proteins via other proteins since both FMDV VP0 and PCBP2 do not display the function of protease. In addition, FMDV 2B inhibits IFN-β signaling pathway by degrading RIG-I proteins^31^; however, the degradation pathways and mechanism are poorly defined. In our study, we observed that FMDV VP0 and PCBP2 degraded VISA through the apoptotic pathway, but the mechanism needs to be elucidated in the future. We also found that FMDV could degrade PCBP2 and VISA; hence, the interaction between PCBP2 and VISA was weakened following FMDV infection.

The coimmunoprecipitation assays indicated that PCBP2 interacted with FMDV VP2, 2B, L, VP0, 2C, and 3D; however, the GST pull-down assay suggested that PCBP2 interacted with FMDV VP2, 2B, VP0, 2C, and 3D. These findings demonstrated that PCBP2 indirectly interacted with the FMDV L protein. Moreover, FMDV 2C, 2B, 3D, and VP0 promoted PCBP2-mediated inhibition of type I interferon; however, only FMDV VP0 markedly enhanced the interaction between PCBP2 and VISA in the degradation of VISA. Further studies are essential to explore the mechanism through which FMDV 2C, 2B, and 3D promote PCBP2-mediated inhibition of type I interferon.

Previous study has demonstrated that PCBP2 induces VISA degradation^17^. Consistent with the previous study, our results showed that FMDV VP0 enhanced the interaction between PCBP2 and VISA and increased PCBP2-mediated degradation of VISA. Based on these findings, we speculated that the interaction between VISA and PCBP2 is more stable in VP0–PCBP2–VISA complex compared to other FMDV proteins-PCBP2–VISA complex. We also found in our study that VISA could inhibit FMDV replication. Because FMDV VP0 enhanced the interaction between PCBP2 and VISA and increased PCBP2-mediated degradation of VISA, FMDV VP0–PCBP2–VISA complex was beneficial to FMDV replication. In addition, FMDV VP0 structural proteins are required for proper assembly of the virus. Therefore, we hypothesized that FMDV VP0–PCBP2–VISA complex was necessary for encapsidation of FMDV.

You et al. unraveled that PCBP2 elicits proteasomal degradation of VISA^17^. Here, we observed that FMDV VP0 and PCBP2 degraded VISA via apoptotic pathways, but not through proteasomal pathway. We speculated that PCBP2 mediated the apoptotic pathway under the regulation of FMDV VP0; however, the mechanism needs to be investigated in future studies. In conclusion, our findings elucidate a unknown mechanism of modulating FMDV by regulating VISA stability via FMDV VP0 and PCBP2.
